# Mucinous borderline ovarian tumors with *BRAF*^*V600E*^ mutation may have low risk for progression to invasive carcinomas

**DOI:** 10.1007/s00404-020-05638-8

**Published:** 2020-06-16

**Authors:** Kaori Ohnishi, Kentaro Nakayama, Masako Ishikawa, Tomoka Ishibashi, Hitomi Yamashita, Kohei Nakamura, Toshiko Minamoto, Kouji Iida, Sultana Razia, Noriyoshi Ishikawa, Satoru Kyo

**Affiliations:** 1 Department of Obstetrics and Gynecology, Shimane University School of Medicine, Enyacho 89-1, Izumo, Shimane, 6938501 Japan; 2 Department of Organ Pathology, Shimane University School of Medicine, Izumo, 6938501 Japan

**Keywords:** *BRAF*, *KRAS*, *TP53*, Mucinous ovarian tumor

## Abstract

**Purpose:**

Mucinous ovarian carcinomas (MOCs) are relatively rare. It has been proposed that a subset of mucinous cystadenomas (MCAs) may progress to mucinous borderline tumors (MBTs), and then to MOCs. *KRAS* is the predominantly mutated gene in MOC; however, other associated mutations and the mechanism underlying carcinogenesis in MOC remain unclear. Here, we assessed molecular genetic alterations in mucinous ovarian tumors and constructed mutation profiles.

**Methods:**

Using the Sanger sequencing method, we assessed genetic mutations (*KRAS*, *BRAF*, *TP53*, and *PIK3CA*) in 16 cases of MOC, 10 cases of MBT, and 12 cases of MCA.

**Results:**

Among MOC cases, the prevalence of G12D and G13D *KRAS* mutations was 43.8% (7/16). No MOC cases showed V600E *BRAF* and *TP53* mutations. Among MBT cases, the prevalence of G12D *KRAS* mutation was 20.0% (2/10), those of *TP53* and *PIK3CA* mutations were nil, and that of V600E *BRAF* mutation was 40% (4/10). None of the genetic mutations assessed were detected among MCA cases.

**Conclusion:**

These results suggest that MBT with V600E *BRAF* mutation may rarely progress to MOC, while MBT with G12D or G13D *KRAS* mutation may more commonly progress to MOC.

## Introduction

Ovarian cancer is the most lethal gynecological malignancy worldwide [1]; recently, its incidence has increased. A dualistic model has been proposed for epithelial ovarian cancer: low-grade disease (type I) develops in a stepwise manner from a benign cystadenoma to a borderline tumor, and then to a carcinoma, whereas high-grade disease (type II) develops de novo from the distal fallopian tube epithelium [2]. Mucinous ovarian tumors can be classified as type I tumors and mucinous ovarian carcinoma (MOC), which is a rare tumor that represents 2–4% of cases of epithelial ovarian carcinoma [3–6]. MOC has a good prognosis if diagnosed at an early stage; however, its prognosis is poor at advanced stages as it tends to be chemoresistant, particularly to platinum drugs [7].

Borderline tumors constitute approximately 10–20% of all epithelial ovarian masses [8]. The most common epithelial borderline tumor in Japan is the mucinous type, while the serous type is the most common in Western countries [9–11]. Ovarian borderline tumors are non-invasive cancers, have a good prognosis, and rarely require systemic therapy.

The RAS-RAF-MEK-ERK-MAP kinase pathway is often implicated in carcinogenesis; particularly, *RAS* oncogenes are key factors in tumor development [12]. *BRAF* and *KRAS* mutations are components of the mitogen-activated protein kinase (MAPK) cascade and *KRAS* mutations are common in mucinous ovarian tumors and prevalent among 40–50% of MOC cases [7]. It has been reported that the rates of *KRAS* mutations in normal ovaries, benign mucinous ovarian tumors, mucinous ovarian borderline tumors, and MOC are 0%, 57%, 90%, and 76%, respectively, suggesting that it may play a major role in the progression from benign tumors to carcinomas [13]. *KRAS* mutation leads to constitutive activation of the protein by increasing guanosine diphosphate/guanosine-5′-triphosphate exchange or by decreasing the guanosine triphosphatase activity of the protein, and thereby associates with constitutive activation of the epidermal growth factor receptor signaling pathway, and brings about increased cell proliferation [12, 14].

The three *RAF* genes (*ARAF*, *BRAF*, and *CRAF*) encode cytoplasmic serine/threonine kinases and are modulated by binding to RAS. *BRAF* mutations brings about ERK activation, which promotes the regulation of the G1/S transition of the cell cycle [12]. *BRAF* mutations were reported in a large proportion of cases of malignant melanoma [15], papillary thyroid cancer [16, 17], colon cancer [17, 18], and hairy cell leukemia [19] with poor outcomes. In contrast, they were reportedly associated with early-stage disease and improved outcomes in patients with low-grade serous ovarian cancer [20, 21]. Thus far, the role of *BRAF* mutations in mucinous ovarian carcinogenesis remains unclear. Additional mutations in mucinous tumors have been observed in *TP53* and *PIK3CA*; however, all these cases emanated from Europe, Australia, or the United States [22–28]. Thus, the carcinogenesis of mucinous ovarian tumor among Japanese patients is still poorly understood. In the present study, we retrospectively investigated the mutation patterns of *BRAF*, *KRAS*, *PIK3CA*, and *TP53* in mucinous cystadenomas (MCAs), mucinous borderline tumors (MBTs), and MOC to clarify the role of each gene in mucinous ovarian carcinogenesis.

## Materials and methods

### Tumor samples

Formalin-fixed, paraffin-embedded tissue samples of 16 MOC, 10 MBT, and 12 MCA patients were used in this study. The samples were retrieved from the Department of Obstetrics and Gynecology, Shimane University Hospital (Izumo, Japan), which have collected from 2008 to 2017. The diagnoses were made based on conventional histopathologic examination of sections stained with hematoxylin and eosin. The tumors were categorized according to the World Health Organization subtype criteria by several pathologists in the Department of Pathology in Shimane University Hospital (Izumo, Japan). The tumors were staged according to the International Federation of Gynecology and Obstetrics classification system. All patients were primarily treated via surgery (i.e., total abdominal hysterectomy, bilateral salpingo-oophorectomy, and omentectomy) with or without pelvic and para-aortic lymph node dissection and adjuvant taxane/platinum combination chemotherapy. The resected specimens of each case were reviewed by a gynecological pathologist (N.I.) and a gynecologic oncologist (K.N.). The protocol for the acquisition of tissue specimens and clinical information was approved by the institutional review board of Shimane University Hospital (Approval No. 2004–0381). All participants provided written informed consent. The study was conducted in accordance with the tenets of the Declaration of Helsinki and Title 45 (United States Code of Federal Regulations), Part 46 (Protection of Human Subjects), effective December 13, 2001.

### Microdissection and DNA extraction

Sixteen MOC, 10 MBT, and 12 MCA cases had sufficient tumor tissue for DNA extraction and sequence analysis. Tissue sections which were reviewed and marked with lines by a skillful gynecological pathologist were placed on membrane slides and counterstained with hematoxylin. Selected tumor tissues on 10-mm sections were dissected under a microscope using a 24-gauge needle to obtain a high percentage of tumor cells. After 48 h of digestion with a proteinase, DNA was extracted from the microdissected samples using a QIAmp DNA Micro Kit (Qiagen, Valencia, CA, USA) according to the manufacturer’s instructions. We have confirmed carcinoma/stroma ratio is more than 50% of each sample.

### Direct sequence analysis

Polymerase chain reaction amplification was performed on exon two of *KRAS*, exon 15 of *BRAF*, exons 4–9 of *TP53*, and exons 9 and 20 of *PIK3CA*, using genomic DNA obtained from microdissected formalin-fixed, paraffin-embedded tissue using the following primers: forward 5′-TTAACCTTATGTGTGACATGTTCTAA-3′, reverse 5′-AGAATGGTCCTGCACCAGTAA-3′ for exon two of *KRAS*; forward 5′-TGCTTGCTCTGATAGGAAAATG-3′, reverse 5′-AGCATCTCAGGGCCAAAAAT-3′ for exon 15 of *BRAF*; forward 5′-CCTGGTCCTCTGACTGCTCT-3′, reverse 5′-GCCAGGCATTGAAGTCTCAT-3′ for exon 4 of *TP53*; forward 5′-TCAGATAGCGATGGTGAGCA-3′, reverse 5′-CTTAACCCCTCCTCCCAGAG-3′ for exon five of *TP53*; forward 5′-TCTGTCTCCTTCCTCTTCCTACA-3′, reverse 5′-AACCAGCCCTGTCGTCTCT-3′ for exon 6 of *TP53*; forward 5′-CTTGGGCCTGTGTTATCTCC-3′, reverse 5′-GGGTCAGAGGCAAGCAGA-3′ for exon seven of *TP53*; forward 5′-GGGAGTAGATGGAGCCTGGT-3′, reverse 5′-GCTTCTTGTCCTGCTTGCTT-3′ for exon 8 of *TP53*; forward 5′-GGAGACCAAGGGTGCAGTTA-3′, reverse 5′-CCCCAATTGCAGGTAAAACA-3′ for exon nine of *TP53*; forward 5′-GGAAAAATATGACAAAGAAAGC-3′, reverse 5′-CTGAGATCAGCCAAATTCAGTT-3′ for exon nine of *PIK3CA*; and forward 5′-CTCAATGATGCTTGGCTCTG-3′, reverse 5′-TGGAATCCAGAGTGAGCTTTC-3′ for exon 20 of *PIK3CA*. All polymerase chain reaction-amplified products were sequenced at Beckman Coulter (Danvers, MA, USA) and analyzed with the Mutation Surveyor DNA Variant Analysis Software (Tokyo, Japan).

### Statistical analysis

All results are expressed as means ± standard deviations. In some cases, the three groups were compared using the chi-square test and the Tukey–Kramer test, as appropriate. All statistical analyses were performed using EZR (Saitama Medical Center, Jichi Medical University, Japan). All differences in analysis items were considered significant at *p* < 0.05.

## Results

**Fig. 1:**
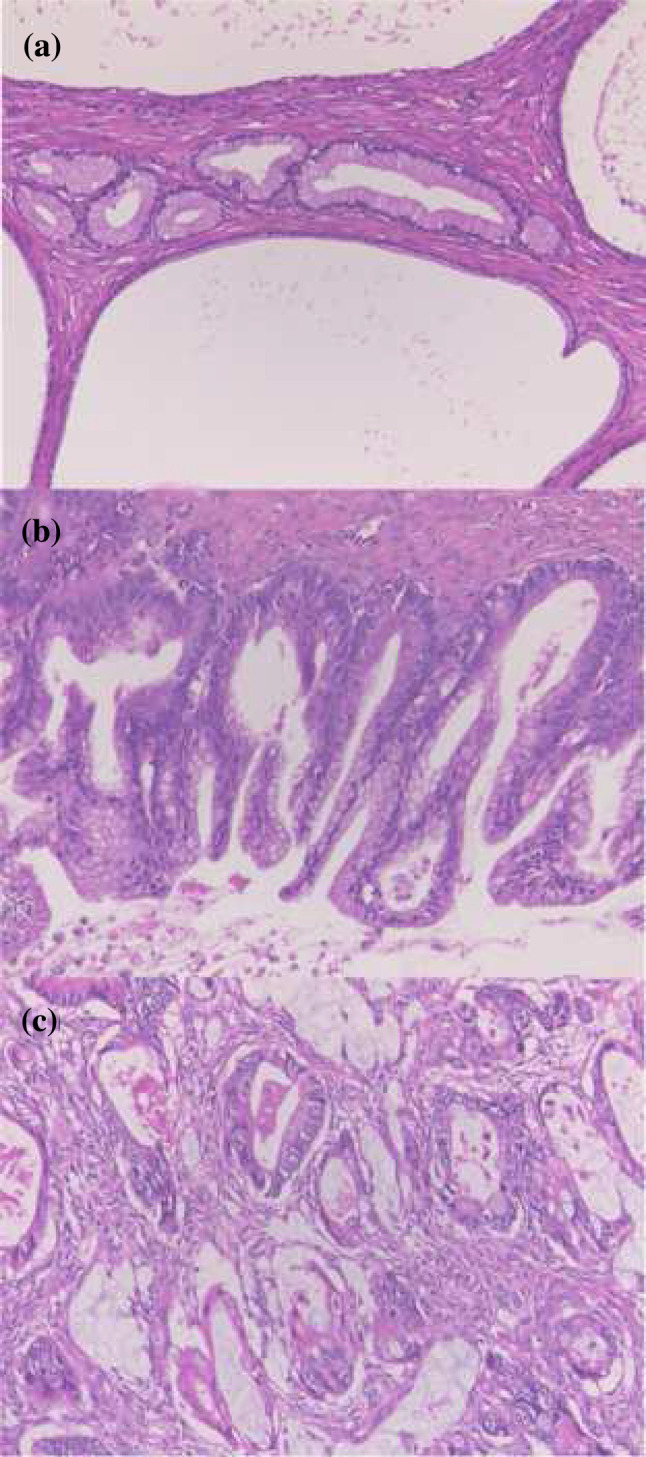
Histological appearance of the mucinous ovarian tumors. (**a**) Hematoxylin and eosin (H&E) staining showing mucinous cystadenoma, (**b**) H&E staining showing mucinous borderline tumor, and (**c**) H&E staining showing mucinous ovarian carcinoma

**Fig. 2:**
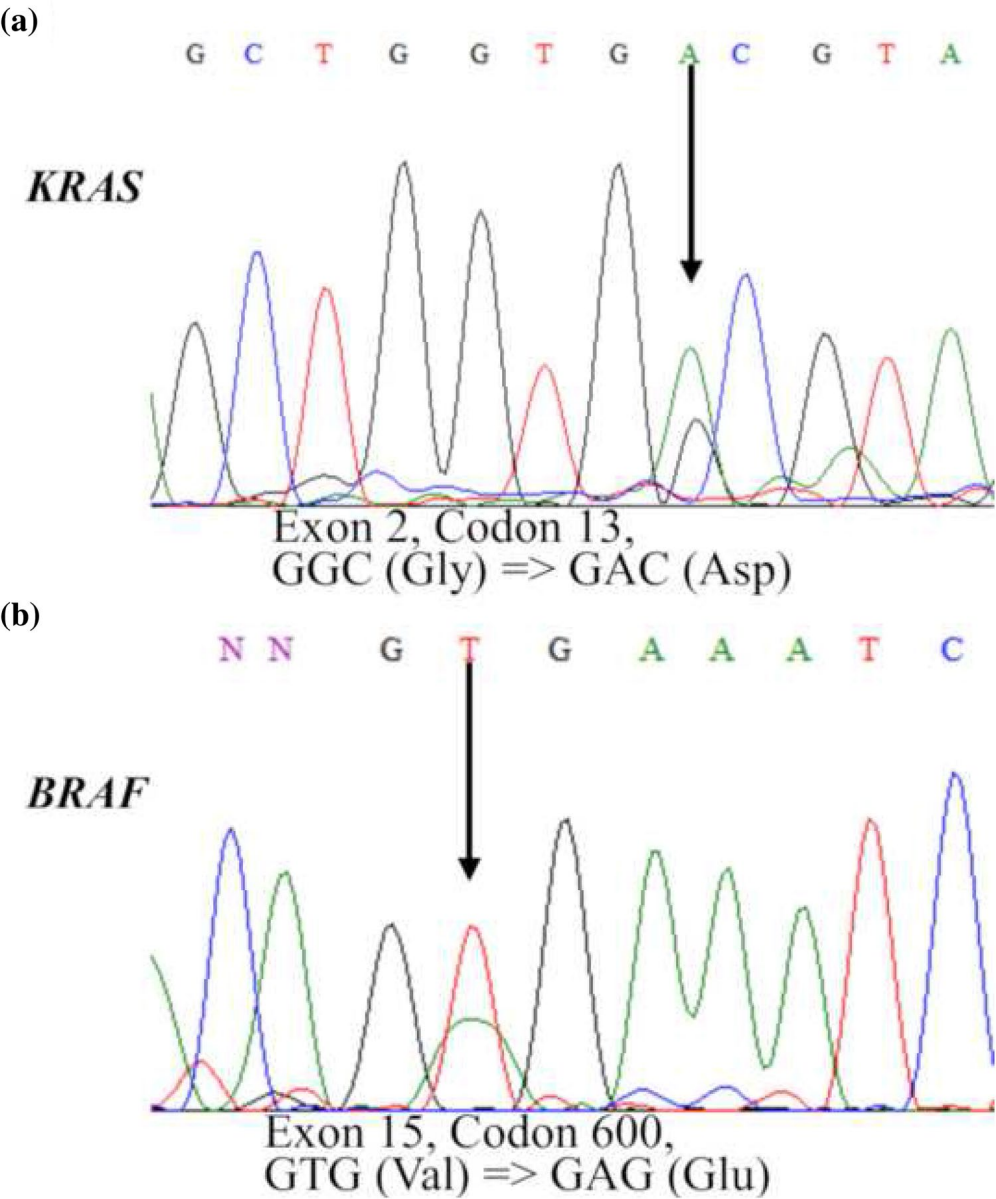
Chromatograms of *KRAS* and *BRAF* mutation statuses in representative ovarian tumors showing (**a**) a point mutation in the *KRAS* gene and (**b**) a point mutation in the *BRAF* gene

To assess the mutation profiles of mucinous tumors of the ovary, we performed direct sequence analysis on 38 tumor specimens, including 16 MOCs, 10 MBTs, and 12 MCAs. The clinical characteristics of the patients are summarized in (Table 1). The mean ages of the patients at diagnosis were 59.6 ± 16.3 years for MOC, 56.5 ± 20.0 years for MBT, and 58.0 ± 18.2 years for MCA. There were no significant differences in the characteristics of the participants, cancer antigen (CA) 125 level, and CA19-9 level. A majority of the patients (81.3%) with MOC were found to have early stage I or II disease at diagnosis, and only 18.8% presented with advanced stage III or IV disease. Figure 1 shows representative examples of the histological appearance of mucinous ovarian tumors. Figure 2 shows typical point mutations in *KRAS* and *BRAF*.

**Table 1:** The number of each mucinous tumors

Histological diagnosis	Carcinoma	Borderline tumor	Cystadenoma	*P value*
Total No. of cases	16	10	12	
Age	59.6 ± 16.3 (25–81)	56.5 ± 20.0 (18–84)	58.0 ± 18.2 (26–80)	
Stage				
I	11	10		
II	2	0		
II	2	0		
IV	1	0		
Early stage (stage I, II)	81.3%			
Advanced stage (stage III, IV)	18.8%			
Grade				
I	8			
II	6			
III	2			
CA125 (U/ml)	121.2 ± 140.2 (8–458)	28.0 ± 20.0 (7–62)	81.9 ± 211.9 (6–749)	0.285*
				0.773^†^
				0.681^‡^
CA19-9 (U/ml)	1913.7 ± 6337.8 (1–24,780)	44.0 ± 56.5 (6–200)	70.2 ± 159.0 (8.3–546)	0.514*
				0.504^†^
				0.999^‡^

*Carcinoma vs. borderline tumor

^†^Carcinoma vs. cystadenoma,

^‡^Borderline tumor vs. cystadenoma (Tukey–Kramer test)

All 38 cases were assessed for mutations in the *KRAS*, *BRAF*, *TP53*, and *PIK3CA* genes. *KRAS* mutations were detected in 7 of the 16 (43.8%) MOC cases and in 2 of the 10 (20%) MBT cases (Table 2). However, no *KRAS* mutations were detected in MCA cases. *KRAS* mutations tended to occur more frequently in MBT than in MCA (*p* = 0.066, Chi square test). *BRAF* mutations in exon 15 were only detected in four of the MBT cases, but not in the MOC or MCA cases. None of the mucinous tumor specimens showed *TP53* mutations. *BRAF* mutations occurred significantly more frequently in MBT cases than in MOC cases (*p = 0.042, Chi square test) (Table 3). *PIK3CA* mutation was detected in only one case of MCA.

**Table 2:** Genetic alterations in mucinous ovarian tumors

No	Hystotype	*KRAS*	*BRAF*	*TP53*	*PIK3CA*
1	Carcinoma	WT	WT	WT	WT
2	Carcinoma	G13D	WT	WT	E545K
3	Carcinoma	G13D	WT	WT	WT
4	Carcinoma	WT	WT	WT	WT
5	Carcinoma	WT	WT	WT	WT
6	Carcinoma	WT	WT	WT	WT
7	Carcinoma	WT	WT	WT	WT
8	Carcinoma	WT	WT	WT	WT
9	Carcinoma	G12D	WT	WT	WT
10	Carcinoma	WT	WT	WT	WT
11	Carcinoma	WT	WT	WT	WT
12	Carcinoma	G12D	WT	WT	WT
13	Carcinoma	WT	WT	WT	WT
14	Carcinoma	G12D	WT	WT	WT
15	Carcinoma	G13D	WT	WT	WT
16	Carcinoma	G12D	WT	WT	WT
17	Borderline tumor	WT	V600E	WT	WT
18	Borderline tumor	WT	WT	WT	WT
19	Borderline tumor	WT	WT	WT	WT
20	Borderline tumor	G12D	V600E	WT	WT
21	Borderline tumor	WT	WT	WT	WT
22	Borderline tumor	WT	V600E	WT	WT
23	Borderline tumor	WT	WT	WT	WT
24	Borderline tumor	G12D	V600E	WT	WT
25	Borderline tumor	WT	WT	WT	WT
26	Borderline tumor	WT	WT	WT	WT
27	Cystadenoma	WT	WT	WT	WT
28	Cystadenoma	WT	WT	WT	WT
29	Cystadenoma	WT	WT	WT	WT
30	Cystadenoma	WT	WT	WT	WT
31	Cystadenoma	WT	WT	WT	WT
32	Cystadenoma	WT	WT	WT	WT
33	Cystadenoma	WT	WT	WT	WT
34	Cystadenoma	WT	WT	WT	WT
35	Cystadenoma	WT	WT	WT	WT
36	Cystadenoma	WT	WT	WT	WT
37	Cystadenoma	WT	WT	WT	WT
38	Cystadenoma	WT	WT	WT	WT

**Table 3:** Frequency of *KRAS* and *BRAF* mutations in mucinous tumors

	KRAS (G12D or G13D)		BRAF (V600E)	
		*P * value		*P * value
PPCarcinoma	7/16 (43.8%)	> 0.05^*^	0/16 (0%)	0.042^*^
Borderline tumor	2/10 (20%)	*0.066*^*†*^	4/10 (40%)	> *0.05*^*†*^
Cystadenoma	0/12 (0%)	> *0.05*^*‡*^	0/12 (0%)	> *0.05*^*‡*^

^*^Carcinoma vs. Borderline tumor

^†^Carcinoma vs. Cystadenoma

^‡^Borderline tumor vs. Cystadenoma (Chi square test)

## Discussion

**Fig. 3:**
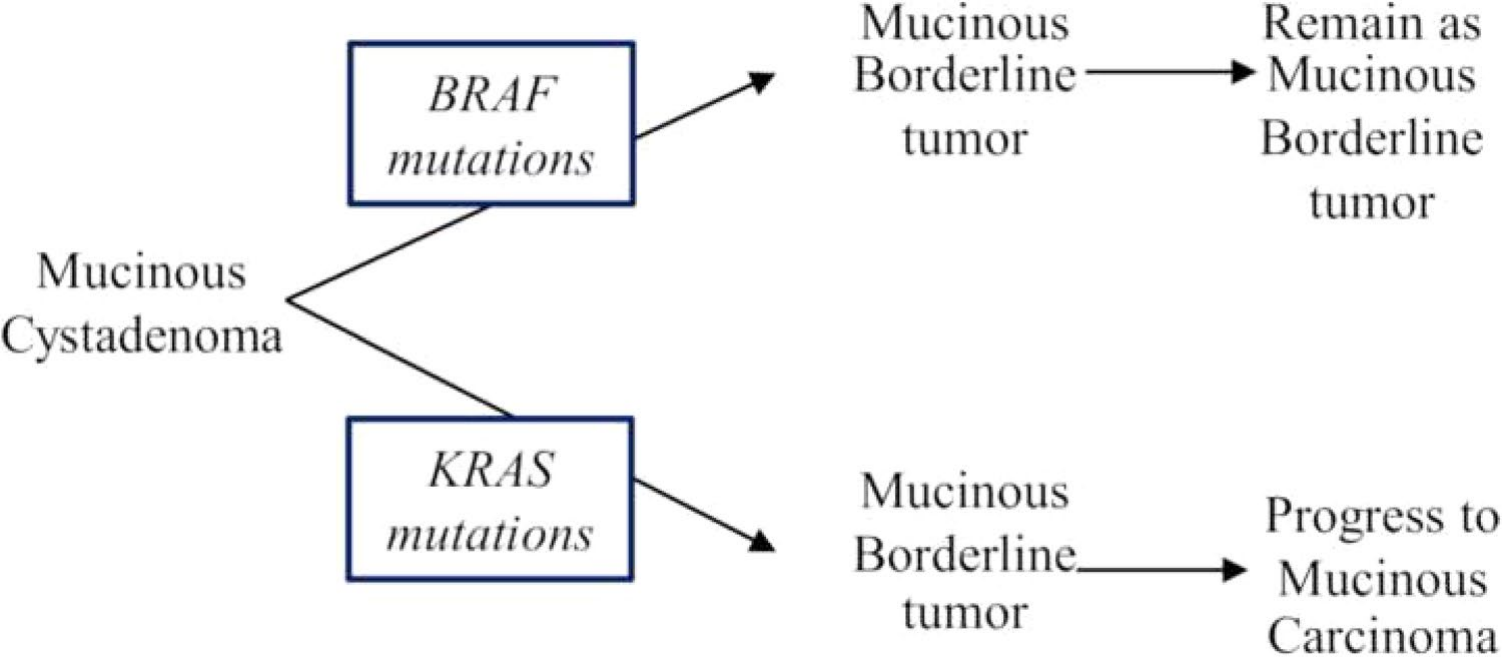
Possible carcinogenic process in mucinous ovarian tumors. Mucinous borderline tumor with *BRAF* mutation may not progress, while mucinous borderline tumor with *KRAS* mutation may progress to invasive carcinoma

In the present study, we performed direct sequence analysis on 38 tumors, including 16 MOC, 10 MBT, and 12 MCA specimens to elucidate the genetic profile of mucinous tumors of the ovary. Interestingly, *BRAF* mutations were more common in MBT than in MOC. However, *KRAS* mutations occurred with high frequency in MOC but with low frequency in MBT. No mutations were detected in the analyzed genes of MCA. These findings indicated that, in the disease continuum from MBT to MOC, the *BRAF* mutation in MBT may not result in progression to MOC, while *KRAS* mutations in MBT may be associated with progression to MOC (Fig. 3).

BRAF is a meaningful serine/threonine kinase that is an element of the RAS-RAF-MEK-ERK signaling pathway and plays a key role in cell proliferation and apoptosis. The complexity of this pathway is increased due to the multiplicity of its components. There are three *RAS* (*HRAS*, *NRAS*, and *KRAS*), three *RAF* (*ARAF*, *BRAF*, and *CRAF*), two *MEK* (*MEK1* and *MEK2*), and two *ERK* (*ERK1* and *ERK2*) genes. They encode proteins and do not have redundant functions [29]. BRAF binds to CRAF and activates its transphosphorylation, thereby regulating the pathway subtly [29].

The V600E *BRAF* mutation constitutes over 90% of all *BRAF* mutations in melanoma [29]. It has been found to activate the MAPK pathway by activating mutations of either *NRAS* or *BRAF* in most melanomas [30]. The BRAF and CRAF protein kinases are the most critical mediators of activated RAS [31]. For mutated *NRAS*, CRAF seems to be important in the downstream activation of MAPKs [32, 33]. RAF interacts with MEK and phosphorylates it, thereby activating ERK [31, 34, 35]. Activated ERK promotes the signal, through altered transcription of several genes [36]. *BRAF* mutations are observed in most melanocytic nevi (70–80%), metastatic melanomas (40–50%), and vertical growth phase melanomas (40–50%) [37–39], and might be an acquired event in early invasive melanoma that induces clonal expansion and tumor progression [36]. Consequently, *BRAF* mutation is associated with poor prognosis in not only melanoma but also papillary thyroid cancer and metastatic colon cancer [15–18]. In contrast, *BRAF* mutations were present in MBT but not in MOC in this study, suggesting that *BRAF* mutations are associated with the indolent type of MBT. Wong et al. reported that *BRAF* mutations are infrequent in advanced-stage low-grade serous ovarian carcinomas and could be improved prognostic markers [20]. Grisham et al. demonstrated that the presence of *BRAF* mutations in serous borderline ovarian tumor or low-grade serous ovarian carcinoma was relevant to early-stage disease and favorable prognoses [21]. Recently, it has been reported that lack of *Cdkn2a* in V600E *BRAF* mutated melanocytes in rodents is associated with rare progression to melanoma [40]. In MOC, *Cdkn2a/b* homozygous deletions/mutations were detected at high frequencies [41]. From these reports, it appears that loss of *Cdkn2a* in mucinous ovarian tumors with V600E *BRAF* mutation impairs progression to carcinoma. Therefore, *BRAF* mutation is associated with early-stage disease, such as MBT, and was not detected in MOC in the present study.

*KRAS* is the predominant mutated gene in MOC and may be related to the progression from benign to malignant tumors [7]. It has been reported that CRAF is a best target for carcinoma with *KRAS* mutations and intensifies MAPK signaling [42, 43]. Our results are consistent with those of previous studies regarding *KRAS*; the prevalences of *KRAS* mutations were 0%, 20%, and 43% among MCA, MBT, and MOC specimens, respectively. We also found that some cases had both *KRAS* and *BRAF* mutations in MBT. These MBT cases with both *KRAS* and *BRAF* mutations might progress to MOC earlier than would those without these mutations.

Recently, it has been reported that *TP53* mutations were key drivers of progression from MBT to MOC [44]. Surprisingly, in the present study, this mutation was not detected in all mucinous ovarian tumors. This discrepancy may have occurred because we investigated only mucinous ovarian tumor specimens obtained from Japanese patients. The carcinogenesis of MOC may be affected by ethnic genetic background. On the other hand, PCR amplification was not performed on exon 2, 3, 10 and 11. There is a possibility that *TP53* mutations could be detected in these exons. Additionally, some MOC cases are high-grade features and they may have *TP53* mutations without exon 4–9.

Our study indicates that *BRAF* and *KRAS* mutations are useful as prognostic biomarkers in MBT patients undergoing surgery. Single *BRAF* mutations in MBT may predict a favorable outcome. However, the patients with *KRAS* mutations might progress to MOC and require careful long-term follow-up.

The present study has several limitations. First, the number of samples in this study is small. This study is ongoing and the number of samples will increase. This will enable us to investigate statistically the relationship between the mutations identified in the present study and patient outcomes. Second, we did not search for loss or mutation of *Cdkn2a* in the present study. In addition, we also need to study *CRAF* mutations in mucinous ovarian tumors. Last, we assessed genetic mutations via Sanger sequencing; therefore, the kinds of gene mutations assessed were limited. Further experimentation with next generation sequencing is necessary to determine details of the molecular mechanism underlying mucinous ovarian carcinogenesis.

In summary, V600E *BRAF* mutations were detected only in MBT, while G12D/G13D *KRAS* mutations were detected more commonly in MOC than in MBT. We posit that MBT with V600E *BRAF* mutation may not progress to MOC and predict a favorable outcome, while MBT with G12D/G13D *KRAS* mutation may progress to MOC in the future.

## Abbreviations

MOC: Mucinous ovarian carcinoma
MBT: Mucinous ovarian tumor
MCA: Mucinous cystadenoma
